# Microwave-Leaching of Copper Smelting Dust for Cu and Zn Extraction

**DOI:** 10.3390/ma12111822

**Published:** 2019-06-05

**Authors:** Behrouz Sabzezari, Seyed Mohammad Javad Koleini, Sina Ghassa, Behzad Shahbazi, Saeed Chehreh Chelgani

**Affiliations:** 1 Department of Mining Engineering, Tarbiat Modares University, Tehran 14115-11, Iran; B.Sabzezari@yahoo.com (B.S.); bzshahbazi@yahoo.com (B.S.); 2 School of Mining, College of Engineering, University of Tehran, Tehran 16846-13114, Iran; 3 Minerals and Metallurgical Engineering, Dept. of Civil, Environmental and Natural Resources Engineering, Luleå University of Technology, SE-971 87 Luleå, Sweden

**Keywords:** copper smelting dust, microwave-leaching, recycling, kinetics, optimization

## Abstract

Industrial wastes may contain high concentrations of valuable metals. Extraction and recovery of these metals have several economic and environmental advantages. Various studies showed positive effects of microwaves as a pretreatment method before the leaching of minerals. However, there are empty rooms for exploring simultaneous microwave and leaching (microwave-leaching) of industrial waste material for the production of valuable metals. This investigation examined the microwave-leaching method to extract copper and zinc from a copper-smelter dust (CSD). The results of microwave-leaching mechanism were compared with conventional heating leaching based on kinetics modelling. The final Cu recovery in the conventional heating and microwave irradiation was 80.88% and 69.83%, respectively. Kinetic studies indicated that the leaching reactions follow diffusion across the product layer. Based on X-ray powder diffraction (XRD) analyses, during conventional experiments sulfate; components formed with high intensity as an ash layer which prevents reagent access to the solid surface and decreases the Cu dissolution. While the sulfate components did not detect in the microwave-leaching residuals which means that microwave irradiation helped to decrease the ash layer formation. Taking all mentioned results into consider it can be concluded that microwave-leaching can be considered as an efficient method for extraction of valuable metals from waste materials.

## 1. Introduction

By decreasing the primary metals resources (ores) and increasing costs of the metals production from minerals, recovery of metals from secondary resources has become a necessity. Secondary metals resources are divided into two main groups: urban and industrial wastes. Extraction of metals from urban wastes include; printed circuit boards [1], batteries [2], magnets [3], liquid crystal displays (LCDs) [4] and etc., was studied in several investigations. Although urban wastes may contain high concentrations of different metals, their extractions can typically be difficult due to their complexity in the collection and sorting. On the other hand, the huge amount of industrial wastes such as slags, catalyst, fly ash, melting dusts and mine tails [5] were produced daily which can be used as an important source for metals production. 

Pyrometallurgical production of copper from mineral concentrates usually contains four steps: melting, converting, refining and electrorefining. A huge amount of dust has been produced during the melting stage as a result of the gas cleaning process [6]. This industrial waste typically contains high concentrations of copper, zinc, lead, iron, arsenic, cadmium and silicon. In one hand, reproduction of these metals can have economic benefits and on the other hand save the environment. Due to high metals concentration and sample complexity, hydrometallurgical methods suggested for recovery of metals from these industrial wastes [7,8,9]. 

As an electromagnetic wave with 0.3 to 300 GHz frequency, the microwave has numerous applications in industries and daily life. These special waves are using extensively in communication, navigation, radar astronomy, spectroscopy, and heating. Since various studies demonstrated that the temperature is an influential parameter in leaching [10,11,12], microwave can be used to provide required heat for leaching. Microwave can increase the temperature of materials in the noncontact system in a short time. The microwave energy adsorption is depended on dielectric constant and dielectric loss factor [13]. Therefore, different materials may have a different temperature in microwave heating, unlike convection heating [14]. 

Various investigations indicated the positive effect of microwaves as a pretreatment technique on leaching kinetics of minerals [15,16] and wastes [17,18]. This method also was used for metals extraction from CSD in few studies. For instance, Xia and Picklesi (2000) investigated the effect of influence parameters on metals extraction from electric arc furnace dust, using microwave energy. [19]. In other study, Turan et al. invested the microwave leaching behavior of blended slag (mixture of converter and flash furnace slag) in presence of H_2_O_2_ and CH_3_COOH as oxidation agents. They showed that a mixture of CH_3_COOH (concentration 4 mol/L) and H_2_O_2_ (concentration 4 mol/L) increase the copper recovery to its maximum (95%).

However, the leaching mechanism under microwave irradiation (microwave-leaching) is vague and the topic of controversy between researchers. For instance, Wen et al. (2017) reported that activation energy of chalcopyrite leaching is affected significantly by microwave heating compared to the conventional heating [14]. They showed that boiling point increase in microwave system which leads to an increase in interfacial reaction temperature and Cu recovery from chalcopyrite. On the other hand, it was reported that extraction of metals increases in microwave-leaching due to the high heating energy which is produced during microwave irradiation. Based on this hypothesis, the microwave heating rate is significantly higher than conventional heating which significantly increases the leaching kinetics [20,21]. Therefore, there is a high potential to use this technique (microwave-leaching) for extraction of metals from industrial wastes.

The main aim of this investigation is to examine microwave-leaching method to recover valuable metals (copper and zinc) from a copper smelter dust. The effect of different parameters (H_2_SO_4_ concentration, solid content, oxidant concentration, microwave power and leaching time) and their interactions were studied by using central composite design (CCD) to find an optimum condition. For comparison purposes, the microwave-leaching mechanism was investigated and compared with conventional heating leaching at optimum conditions based on kinetics modelling.

## 2. Materials and methods

### 2.1. Sample Preparation and Characterization

**Figure 1:**
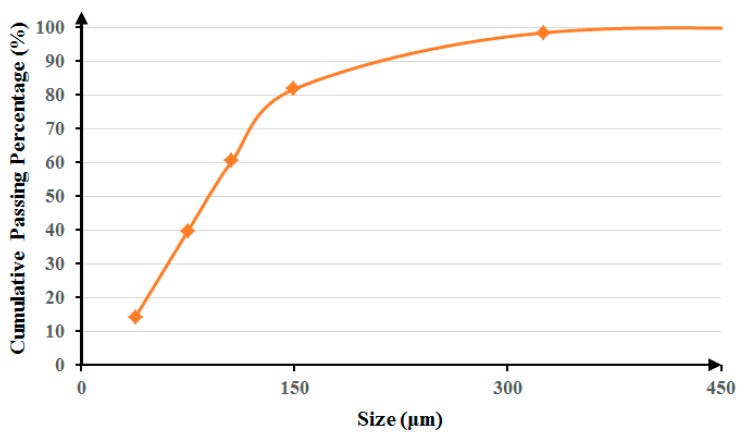
Copper smelter flue dust size distribution.

To provide a representative sample, 50-kg copper smelting dust (CSD) was collected during several weeks from a copper smelting plant in Tehran, Iran. To make the sample dry (the CSD contained 0.28% moisture), it was heated for 24 h at 100 °C. The size distribution analysis indicated that the d_80_ of the sample is 150 μm (Figure 1).

**Figure 2:**
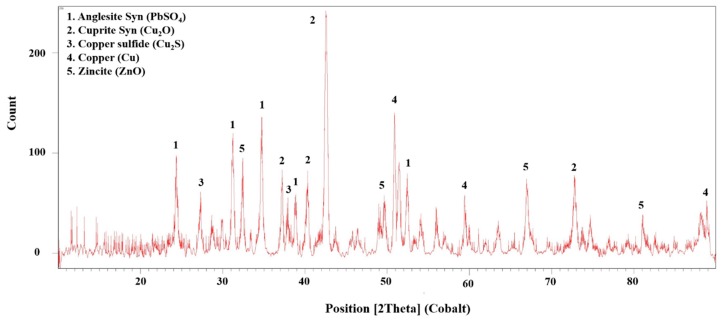
XRD diffractogram of copper smelter flue dust.

X-ray powder diffraction (XRD, D8-Advance, Bruker axs, USA) analyses (Figure 2) showed that the source of copper in the samples are chalcocite (Cu_2_S), cuprite (Cu_2_O) and native copper (Cu). The CSD also contained zincite (ZnO) and anglesite (PbSO_4_).

0.5g CSD was digested in hot HNO_3_ in a sand bath and analyzed with an atomic absorption spectrometer (Varian-AA240, Australia). The copper and zinc concentrations were 65.52% and 4.15%, respectively (the mean of 6 times analysis). Other metals concentrations were analyzed with X-ray fluorescence (XRF, TX2000, GNR, Italy) and atomic absorption spectrometer (ASS) (Table 1). 

### 2.2. Reactor

**Figure 3:**
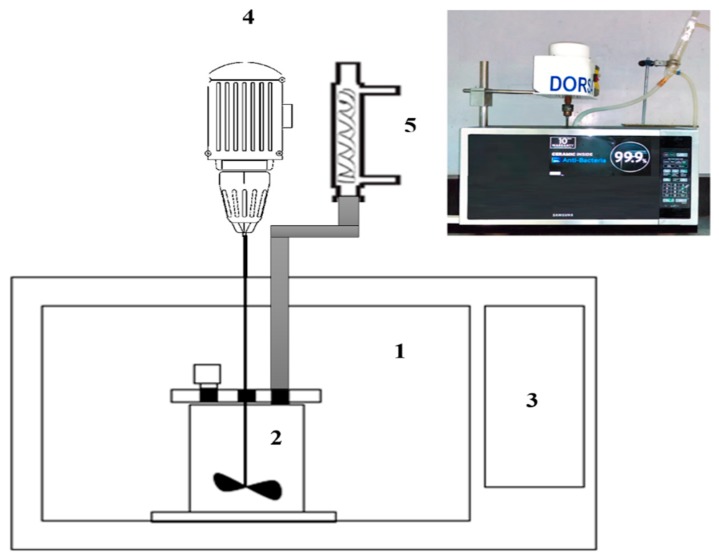
The microwave-leaching set-up (**1**: microwave chamber; **2**: Teflon reactor; **3**: controller; **4**: mixer; **5**: condenser reflux).

The microwave was radiated during the leaching process. A reactor heart (a Teflon cylinder with 8 cm inside diameter and 11 cm height) was located inside a SAMSUNG-ME6194ST microwave (Klang, Malesia) with power control (from 100 to 1000 W). The microwave capacity was 54 L with a triple wave distribution system. The Teflon was selected because this material has no wave adsorption and waves transfer without any drop. The cylinder had a completely sealed cap to avoid evaporation, with three holes for sampling, entering mixer (Rhymebus, Taiwan) and reflux condenser. Two holes also made on top of the microwave for entering mixer and condenser (Figure 3). 

### 2.3. Leaching Procedure

#### 2.3.1. Oxidant Presentation

The sulfide minerals (such as chalcocite) have a very low solubility on sulfuric acid. Thus, it would be essential to use an oxidant for increasing recovery of metals. Nitric acid (HNO_3_) was selected as an oxidizing agent for the CSD dissolution. To clarify the effect of oxidation agent, two tests without any oxidant were performed at 150 and 300 g/L sulfuric acid concertation and results compared with two tests with 10 and 30 g/L HNO_3_. All tests were performed in 5% pulp density, 300 rpm agitation rate, 300 mL leaching reagent with 150 g/L H_2_SO_4_ and 1000 W microwave power. Processing time was set on 30 min for all tests. The Cu and Zn recoveries were calculated by analyzing Cu content in the pregnant leach solution (PLS) after each test.

#### 2.3.2. Leaching Experiments

Response surface methodology (RSM) and central composite design (CCD) were employed to study the effect of leaching parameters (acid concentration, pulp density, oxidant concentration, microwave power and leaching time) on copper and zinc dissolution. RSM is a statistical experimental design method for process modelling and optimization. The effect of each parameter and their interaction can be determined by RSM with minimum number of experiments [22]. The results of experiments are achieved from CCD can be described according to the following equation [23]:

(1) $$Y=b_{0}+\overset{k}{\underset{i=1}{\sum }}b_{i}X_{i}+\overset{k}{\underset{i=1}{\sum }}b_{ii}X_{i}^{2}+\overset{k}{\underset{i;j=1\;\left(i\neq j\right)}{\sum }}b_{ij}X_{i}X_{i}$$

where *Y* is the response, *b*_0_ is constant coefficient, and *b_i_*, *b_j_*, and *b_ij_* are linear, quadratic and interaction coefficients. *X_i_* and *X_j_* are the coded value of independent parameters. *X_i_X_j_* and *X_i_*^2^ show the interaction and quadratic terms. Analysis of variance (ANOVA) can be used to validate a mathematical model based on null-hypothesis. The higher *F*-value and lower *p*-value (indexes which are calculated based on Fisher test) indicate more significant model. In general, 32 microwave-leaching experiments were designed. The investigated parameters and their levels were listed in Table 2. 

CCD was developed by Box and Wilson in the 1950s [24] and used extensively for chemical process optimization in recent decades [25,26]. This experiment design consists three following parts: (1) a full factorial design, (2) a star design in which experimental points are at α distance from its center and (3) center point [27]. This means that CCD can investigate the effect of parameters in 5 levels. Typically, the center point is repeated several times to predict the errors and experiment repeatability. The center point test (acid concentration = 150 g/L, pulp density = 15%, oxidant concentration = 20 g/L, microwave power = 600 W and leaching time = 10.5 min) was repeated 6 times to determine the experiments repeatability. The recovery of copper and zinc extraction were selected as responses. 

#### 2.3.3. Kinetics Study

Kinetics modelling is a strong tool to clarify the leaching mechanism. Generally, the leaching process contains two main steps in series: (1) the escape of solute molecules from the solid surface, (2) the diffusion of these molecules toward the bulk liquid phase [11]. In other words, the leaching process is controlled by chemical or diffusion parameters or combinations of these two mechanisms. Different kinetics models have been developed based on this idea. The kinetics models which are common for description of leaching mechanism have been listed in Table 3. 

Thus, kinetics study was performed to determine the leaching mechanisms in presence and absence of microwave radiation. The microwave-leaching kinetics test carried out at the optimum conditions based on leaching experiment results. Process followed for 10 min and 7 samples withdrawn from the reactor at different intervals. Furthermore, the kinetics tests examined for non-microwave-leaching tests at 90 °C, to explore the leaching mechanism in the absence of microwave. For these tests, a Teflon reactor located in a water-bath (N-Biotek) to control the temperature. All other conditions for these two kinetic tests were completely the same. Different kinetics models (Table 3) have been fitted to the provided data to identify the leaching mechanism. 

## 3. Results and Discussion

### 3.1. Oxidant Presentation

Oxidizing agents can be added directly to the leaching reactor. To show the effect of oxidizing agent on Cu extraction, four pre-tests were carried out (Table 4). The copper recovery in tests without oxidizing agent is negligible. Increasing the sulfuric acid concertation from 150 to 300 (g/L) slightly improves the Cu recovery. On the other hand, addition of nitric acid significantly increases the Cu recovery. Increasing the nitric acid concertation shows an improvement in the Cu dissolution. Therefore, the HNO_3_ concentration should be investigated as one of the effective parameters on the leaching process.

### 3.2. Process Optimization

For the process optimization, the effect of five parameters (H_2_SO_4_ concertation, solid content, microwave power, oxidizing agent concertation and process time) and their interactions have been investigated by using CCD of RSM. Responses of various microwave-leaching conditions based on CCD have been shown in Table 5.

#### 3.2.1. Copper Extraction

The copper recovery rate in function of five mentioned parameters have been modeled and expressed in Equation (2). The variance analysis (ANOVA) carried out to determine the model accuracy (Table 6). ANOVA is a statistical tool to specify the significance of data based on Fisher ratio of variances [26,36] by calculation of *F* and *p* values [37]. The *F*-value should be maximized and *p*-value should be minimized to achieve a significant statistical model. The suggested model (Equation (2)) is completely significant with high *F*-value (26.15) and low *p*-value (less than 0.0001) which means that there is only 0.01% chance that model responses occur due to the noise [38]. The model coefficient of determination (R^2^) is 0.9150 which shows well agreement between predicted and actual data. Adequate precision shows a signal-to-noise ratio of 18.784.

(2) $$\begin{array}{l} \frac{1}{R_{Cu}}=0.0236+\left(1.8295\times 10^{-4}\right)A+\left(2.4602\times 10^{-3}\right)B-\left(3.3729\times 10^{-6}\right)C-(9.8987\times \\ 10^{-5})D-\left(1.4136\times 10^{-3}\right)E-\left(3.9523\times 10^{-6}\right)AB+\left(4.9518\times 10^{-5}\right)BE-(6.4229\times \\ 10^{-7})A^{2}-\left(5.1155\times 10^{-5}\right)B^{2} \end{array}$$

**Figure 4:**
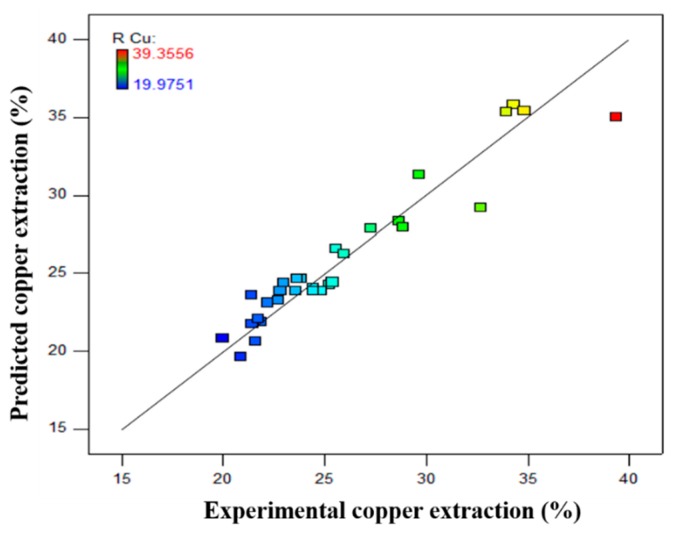
Predicted vs. actual plots for copper recovery.

The predicted data generated from Equation (2) versus the data achieved from experiments (actual data) have been shown in Figure 4. The accuracy of the correlation between predicted and actual data confirms due to the position of the points on both sides of the 45° line [39]. As mentioned, the center point test repeated six times to calculate the error and determined the tests repeatability. The calculated pure error was 9.987 × 10^−6^ which shows a negligible error in the experiments. Lack of fit is an index to compare the residual and pure error from the replicated design points. The higher level of the *p*-value for lack of fit indicates higher tests repeatability and should be not-significate (higher than 0.005). The lack of fit for the suggested model (Equation (2)) is 0.1324 which is “not-significant”. Therefore, experiments have a high repeatability rate.

**Figure 5:**
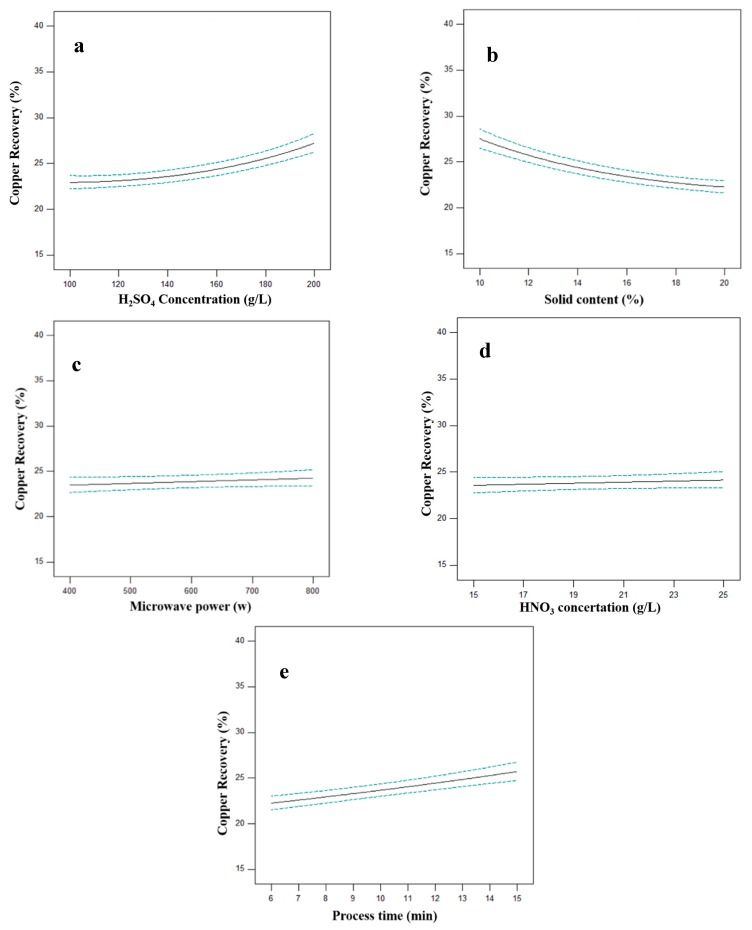
Effect of sulfuric acid concentration (**a**), solid content (**b**), microwave power (**c**), HNO_3_ concentration (**d**) and process time (**e**) on Cu recovery (other parameters are held at center level).

The effects of parameters on copper recovery were depicted in Figure 5. In this diagram, a parameter changed while all other parameters held at their center levels. In general, the effect of each parameter can increase by increasing in diagram slope. According to Figure 5c,d, microwave power and oxidizing agent concentration have a negligible effect on copper recovery, at the selected interval. This means that by increasing in these parameters the energy and chemicals consumption will increase without any significant improvement in the extraction of Cu from CSD. On the other hand, sulfuric acid concertation, solid content and processing time have a meaningful influence on copper dissolution. The acid concertation and leaching time have a direct effect on process efficiency which means their increase can improve the Cu recovery (Figure 5a,e). While increasing solid content, the copper recovery shows a decrease (Figure 5b). In fact, by increasing the pulp density (in constant leaching reagent concentration) the acid diffusion to particles surface will decrease which cause a decline in metals dissolution. 

The ANOVA also can be used to determine the importance of parameters. The parameters with higher *F*-value and lower *p*-value have a more significant effect on a model [40]. With a *p*-value lower than 0.0001, acid sulfuric concentration, solid content and process time have a more significant effect on copper recovery. 

**Figure 6:**
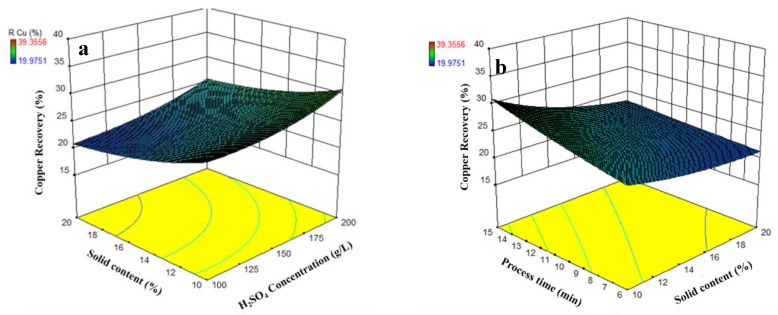
The solid content-H_2_SO_4_ concentration (**a**) and process time-solid content (**b**) interactions (other parameters are held at center).

According to Equation (2) and Table 6, there are sulfuric acid concentration-solid content and solid content-process time interactions. The interactions between parameters are shown in Figure 6. These results indicate that increasing the H_2_SO_4_ concentration and decreasing solid content simultaneously lead to a higher copper recovery. In addition, the highest Cu recovery occurs by increasing the leaching time and decreasing solid content. During the leaching process, the chemical reagent should diffuse to the particles surface and react to dissolve the metals. This means that metals recovery is dependent on reagent concentration and particles surface (which is a function of solid content) [41]. By increasing the H_2_SO_4_ concentration and decreasing copper smelting dust in leaching environment a higher amount of acid can diffuse to the unit of CSD surface, which improve the leaching efficiency. This reason also can be used to explain the interaction of time and solid content. The specific surface area will increase by increasing in solid content and this cause an increase in reaction time. Therefore, higher time requires for reaction in higher pulp density. 

Based on Equation (2), the optimum condition to achieve the highest Cu recovery is H_2_SO_4_ 250 (g/L), solid content 5 (%), microwave power 1000 (W), HNO_3_ 10 (g/L) and leaching time 10 (min). It should be noted that nitric acid concertation has no effect on Cu recovery in the selected intervals. Therefore, its optimum condition selected in its lowest concertation (10 g/L) to decrease the chemical consumption and corrosion. 

#### 3.2.2. Zinc Extraction

The effects of five mentioned parameters also have been investigated in zinc dissolution. Equation (3) has been suggested to predict Zn recovery. The analysis of variance for the suggested model is reported in Table 7. This model is significant with *p*-value = 0.001. The adequate precision for this model is 7.772. The adequate precision for the model is higher than 4 (the minimum required amount). The lack of fit for this model is not-significant which shows that repeatability of experiments for zinc extraction.

(3) $$\begin{array}{l} R_{Zn}=0.6954+\left(0.4189\right)A+\left(0.7899\times 10^{-3}\right)B+\left(6.9610\times 10^{-4}\right)C+\left(1.0263\right)D+\\ \left(0.1266\right)E+\left(2.9574\times 10^{-3}\right)BC-\left(2.6764\times 10^{-3}\right)CD-\left(1.4265\times 10^{-3}\right)A^{2}-\left(0.0952\right)B^{2}. \end{array}$$

**Figure 7:**
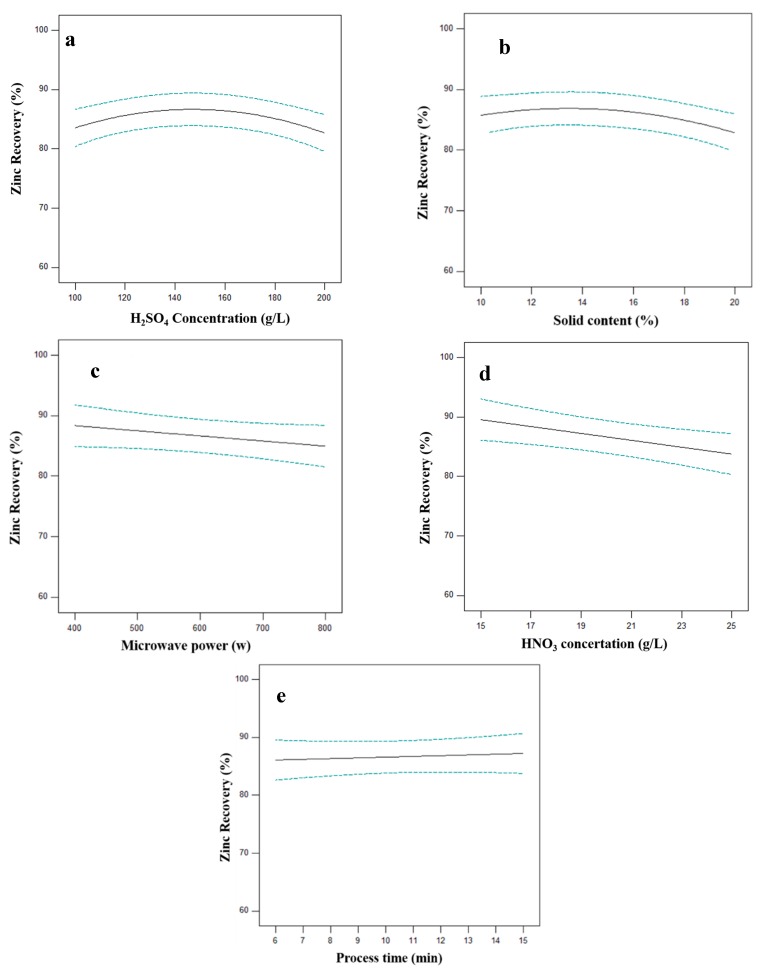
Effect of sulfuric acid concentration (**a**), solid content (**b**), microwave power (**c**), HNO_3_ concentration (**d**) and process time (**e**) on Zn recovery (other parameters are held at center level).

The effects of each parameter on Zn recovery are shown in Figure 7. As mentioned, the effect of parameters also can be determined by ANOVA. According to Table 3 and Figure 7, the HNO_3_ concentration has the highest effect on zinc dissolution. The nitric acid concentration has a higher effect on Zn recovery compare to sulfuric acid concentration. It should be noted that nitric acid does not act as an oxidizing agent for ZnO because zincite is an oxide component and does not need any oxidizer for dissolution. This means that HNO_3_ act as a leaching agent for ZnO. With *F*-value 0.32 microwave power has a negligible effect on zinc leaching. Therefore, ZnO dissolution is chemical leaching rather than microwave leaching. The extending leaching time also has no influence on zinc leaching improvement. Despite the copper composition, the zinc present in the sample in the form of oxide component which is highly soluble in acid [42] with high leaching kinetics. In fact, almost all ZnO dissolved in the first minute of the process. 

**Figure 8:**
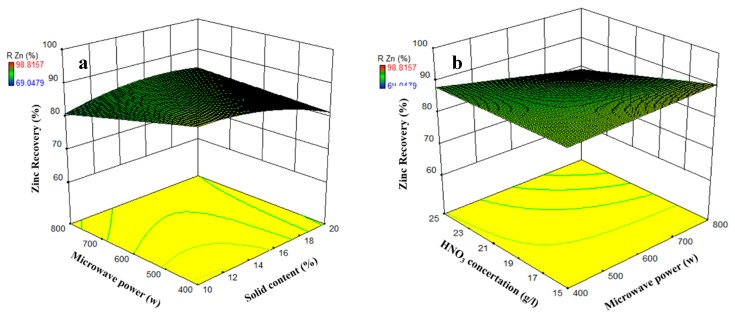
The Microwave power-solid content (**a**) and HNO_3_ concentration-microwave power (**b**) interactions (other parameters are held at center level).

Among all interactions between parameters, just microwave power-solid content and microwave power-HNO_3_ concertation interactions are meaningful (with a *p*-value lower than 0.05). The effects of these interactions on Zn recovery are shown in Figure 8. The zinc recovery becomes its maximum amount by minimizing microwave power and solid content, simultaneously (Figure 8a). On the other hand, the Zn recovery will be minimized by maximizing the microwave power and minimizing nitric acid concertation (Figure 8b). The drawn surfaces (Figure 8b) have a slight curve that shows the minor effect of detected interactions. 

The optimum H_2_SO_4_ and HNO_3_ concentrations for Zn dissolution is 150 g/L and 10 g/L, respectively. The microwave power and pulp density should fix on 1000 W and 25% to achieve the highest Zn recovery in the highest capacity (due to high solid content). The time also has no effect on Zn leaching process in the selected period, which means that optimum leaching time can be selected at 6 min. As mentioned, ZnO dissolves in acid with high kinetics due to its oxide composition.

### 3.3. Kinetics Study

**Figure 9:**
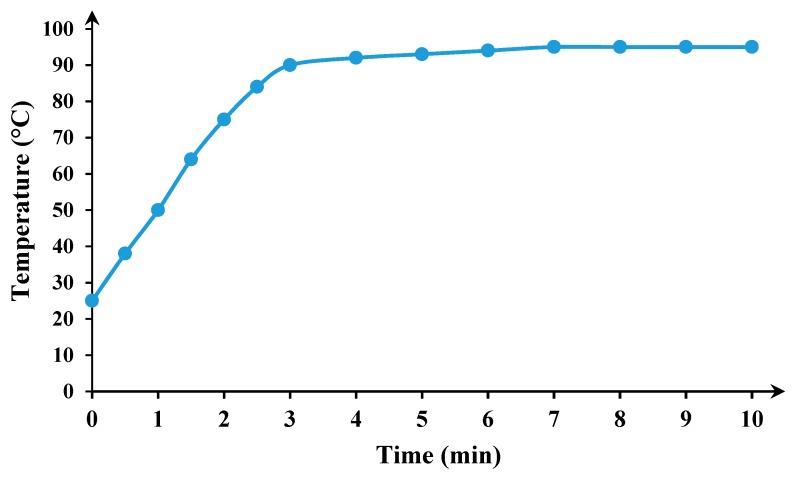
Leaching agent temperature fluctuation during microwave irradiation with 1000 W power.

As mentioned above, Zn dissolution occurs quite quickly; thus, sampling for the kinetic study assessment is not possible. However, the kinetic study carried out to determine the Cu leaching mechanism from CSD, in the presence and absence of microwave irradiation. For comparison purposes, it would be essential to operate the experiments in the same condition. Therefore, all conditions include leaching and oxidizing agent concentrations, solid content and process time sat on optimum conditions achieved from the optimization section. To find the temperature during microwave leaching, the leaching liquid temperature was measured for 1000 W microwave power. Figure 9 shows the temperature fluctuations during 10-min irradiation. According to this diagram, the liquid temperature started from ~25 °C (room temperature) and reached its highest level (~90 °C) in 3 min. To provide similar conditions, non-microwave leaching test also carried out in a water bath at a temperature of 90 °C.

**Figure 10:**
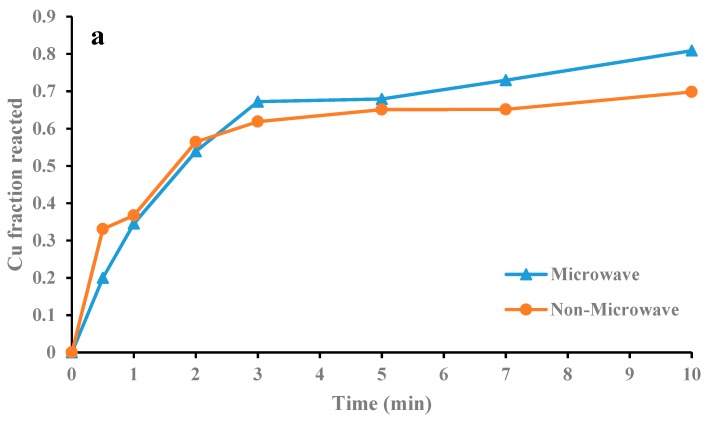

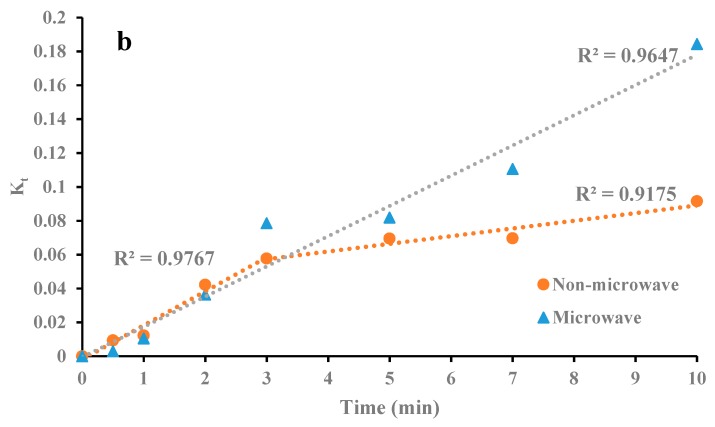
Copper extraction (**a**) and plot of K_t_ versus time for copper leaching in presence and absence of microwave irradiation (**b**).

Figure 10a shows the Cu recovery in the microwave and non-microwave leaching. The final Cu recovery from CSD after 10 min in the absence and presence of microwave irradiation is 80.88% and 69.83%, respectively. The copper dissolution in non-microwave leaching is higher in the first 3 min; however, for microwave-leaching experiment Cu dissolution increases after this period. The non-microwave leaching was carried out in the water bath and the reaction temperature was constant during the process. On the other hand, the temperature reaches its highest level after 3 min (Figure 9). After this initial step, the Cu dissolution in the microwave-leaching experiment starts to increase (compare to non-microwave leaching). 

**Figure 11:**
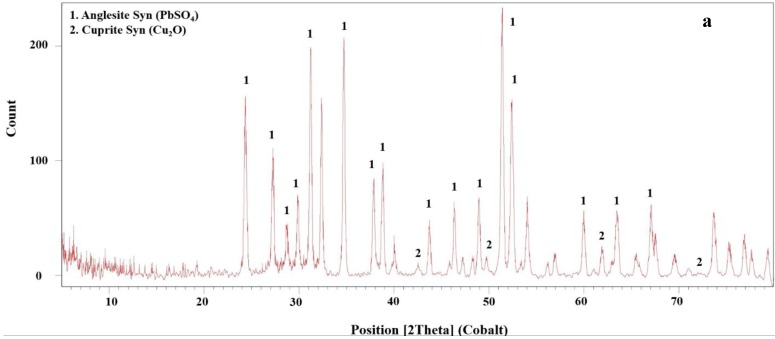

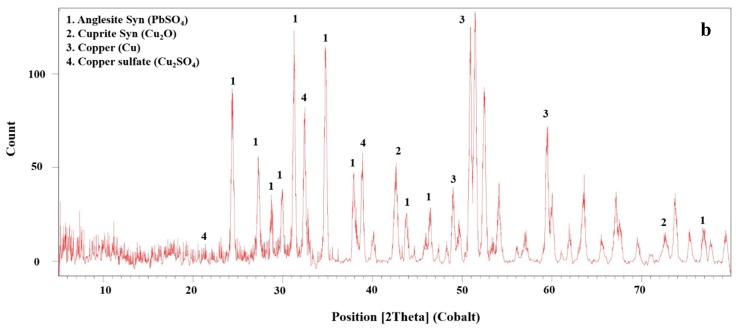
XRD diffractogram for microwave leaching (**a**) and non-microwave leaching (**b**) residuals.

Different kinetics models (Table 3) have been fitted to data to find the best mechanism to describe the leaching process. The $k_{t}=\frac{1}{3}\ln\left(1-x\right)+{\left(1-x\right)}^{-\frac{1}{3}}-1$ model was the best-fitted model for data achieved from both microwave and non-microwave leaching (Figure 10b). This model shows that the leaching reaction follows interfacial transfer and diffusion across the product layer [32]. The kinetics modelling indicated that non-microwave leaching follows two stops. Although both septs are fitted to the above mentioned equation, the reaction speeds for different steps are varied. The reaction kinetics for the first 3 min is high and drops due to the formation of the product layer. Leaching residuals are analyzed by the XRD to determine the composition of the product layer during leaching (Figure 11). The microwave-leaching residual contains Cu_2_O; while the non-microwave leaching residual contains Cu_2_SO_4_, Cu_2_O, and metallic Cu. The Cu_2_O intensity in microwave-leaching residual is very low; therefore, the quantity of this component is not significant. The sulfate component intensity, on the other hand is high for non-microwave leaching. According to the kinetics modeling and XRD results, the formation of sulfate components limited Cu dissolution for non-microwave leaching experiments. The leaching reagent should diffuse across this product layer to access the surface of particles for leaching. 

**Figure 12:**
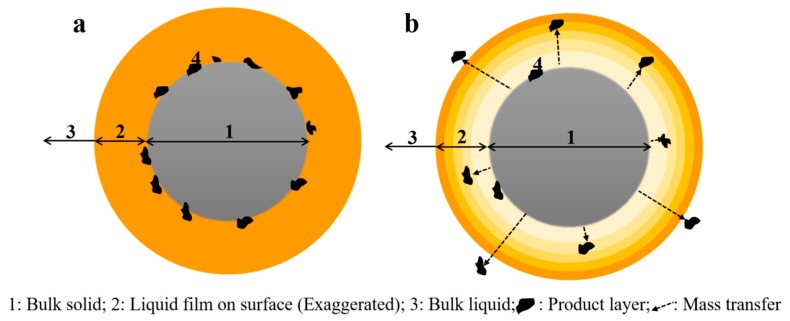
Product layer covers the surface and prevent liquid access to surface in convection-leaching (**a**); The temperature gradient on liquid film causes a mass transfer and reduce the ash layer formation in microwave-leaching (**b**).

The ability of materials for microwave energy adsorption is dependent on dielectric properties [43]. Therefore, microwave adsorptions for solid and liquid are quite different and increasing temperature for these two phases has to be varied. These variations make a thermal gradient in the solid-liquid boundary (liquid film). This thermal gradient causes a mass transfer in particles surface and avoids ash layer formation. Therefore, the leaching kinetics increase in microwave-leaching due to higher reagent diffusion to the surface by decreasing the ash layer formation (Figure 12). 

Two additional experiments without agitation also carried out to clarify the leaching mechanism, in the presence and absence of microwave irradiation. The Cu recovery under microwave irradiation was 63.99%; while its recovery was 23.44 in absence of microwave. These tests also show the positive effect of microwave irradiation on leaching kinetics. The microwave power increases interactions between molecules, produce heat and improve the kinetic energy [16]. The lead sulfate (anglesite) determines in both microwave and non-microwave leaching residual. With a K_sp_ = 1.62 × 10^−8^ [44,45], PbSO_4_ remained unreacted in the sample during the leaching process.

## 4. Conclusions

Leaching under microwave irradiation (microwave-leaching) was examined for Cu and Zn extraction for copper smelting dust (CSD) to produce valuable metals and reduce environmental problems of this waste component. The high efficiency, fast kinetic and low waste of energy are the most important advantages of this method compared to the traditional methods. Response surface methodology (RSM) and central composite design (CCD) were used to design various experiments based on leaching parameters (acid concentration, pulp density, oxidant concentration, microwave power and leaching time) and find optimum conditions for copper and zinc extraction. CCD results indicated that microwave power and oxidizing agent concentration do not show a significant effect on copper extraction while there is a correlation between sulfuric acid concertation, solid content, processing time and copper dissolution, in selected intervals. In other words, increasing the H_2_SO_4_ concentration and decreasing solid content simultaneously can increase copper recovery. Moreover, the highest Cu extraction occurred by increasing the leaching time and decreasing solid content. The optimum condition to receive the highest Cu recovery was: H_2_SO_4_ 250 g/L, solid content 5%, microwave power 1000 W, HNO_3_ 10 g/L and leaching time 10 min. For Zn recovery, the HNO_3_ concentration has the highest effect. HNO_3_ which used as an oxidant for Cu recovery acted as a leaching agent for ZnO. Zinc oxides immediately dissolve in the acids, and the Zn recovery reached its maximum percentage when at H_2_SO_4_ 150 g/L, HNO_3_ 10 g/L, microwave power of 1000 W and pulp density of 25%. 

To better understand the mechanisms of Cu leaching from CSD and for comparison purposes, various kinetics models are fitted to the data resulted from experiment design, in the absence and presence of microwave. The final Cu recovery from CSD in the absence and presence of microwave irradiation was 80.88% and 69.83%, respectively. Kinetics studies demonstrated that non-microwave leaching follows two steps: (1) High Cu dissolution rate (in first 3 min), (2) low Cu dissolution rate (after first 3 min) due to the formation of a product layer. Moreover, they indicated that the leaching reaction follows interfacial transfer and diffusion across the product layer. XRD analysis showed that microwave-leaching residual contains low concertation of Cu_2_O, while non-microwave leaching residual contains a high concentration of Cu_2_SO_4_. In other words, the sulfate component intensity was high on the surface of particles for non-microwave leaching which decreased Cu dissolution for non-microwave leaching experiments. While microwave irradiation decreases the ash layer formation and increases the Cu dissolution. 

## Figures and Tables

**Table 1:** Chemical composition of copper smelting dust.

**SO_3_ (%)**	**Pb (%)**	**SiO_2_ (%)**	**Na_2_O (%)**	**Al_2_O_3_ (%)**	**Fe_2_O (%)**	**CaO (%)**	**As (%)**	**Cl (%)**
14.69	13.13	7.56	6.55	2.73	2.25	1.41	0.92	0.77
**K_2_O (%)**	**Sn (%)**	**Sb (%)**	**Bi (%)**	**Ni (%)**	**I (%)**	**Ag (ppm)**	**Cd (ppm)**	**Co (ppm)**
0.52	0.38	0.29	0.23	0.13	0.11	84.80 *	55.65 *	33.40 *

* Measured with AAS.

**Table 2:** The examined parameters and their level.

Parameter	Levels				
	1	2	3	4	5
H_2_SO_4_ concentration (g/L)	50	100	150	200	250
Solid content (%)	5	10	15	20	25
Oxidant concentration (g/L)	10	15	20	25	30
Microwave power (W)	200	400	600	800	1000
Leaching time (min)	1.5	6	10.5	15	19.5

**Table 3:** Kinetics models suggested for leaching process (X = fraction reacted, K_t_ = kinetic constant, t = time).

Model	Mechanism	References
$k_{t}=1-{\left(1-X\right)}^{\frac{1}{3}}$	Chemical reaction control	[28]
$k_{t}=1-\frac{2}{3}X-{\left(1-X\right)}^{\frac{2}{3}}$	Diffusion control	[28]
$k_{t}=1-{\left(1-0.45X\right)}^{\frac{1}{3}}$	Surface chemical reaction by shrinking core model	[29]
$k_{t}={\left[1-{\left(1-X\right)}^{\frac{1}{3}}\right]}^{2}$	Diffusion through product layer	[30]
$k_{t}=1-\frac{2}{3}X-{\left(1-X\right)}^{\frac{1}{3}}$	Diffusion through a porous product layer by shrinking core model	[31]
$k_{t}=\frac{1}{3}\ln\left(1-X\right)+(\left(1-X{)}^{\frac{1}{3}}-1\right)$	Interfacial transfer and diffusion across the product layer	[32]
$k_{t}=1-3{\left(1-X\right)}^{\frac{2}{3}}+2\left(1-X\right)$	Diffusion of hydrogen ions through a product layer by shrinking core model	[33]
$k_{t}=1-{\left(1-X\right)}^{\frac{2}{3}}$	Mixed control model by shrinking core model (diffusion control; chemical reaction control)	[34]
$k_{t}=-\ln\left(1-X\right)$	Mixed control model (surface reaction control; and diffusion through sulfur layer)	[35]
$k_{t}=\frac{1}{5}{\left(1-X\right)}^{-\frac{5}{3}}-\frac{1}{4}{\left(1-X\right)}^{-\frac{4}{3}}+\frac{1}{20}$	Mixed control model based on reactant concentrations	[36]

**Table 4:** Effect of oxidizing agent on copper recovery from CSD.

No.	H_2_SO_4_ Concertation (g/L)	Oxidizing Agent	Oxidizing Agent Concertation (g/L)	Cu Recovery (%)
1	150	-	-	17.97
2	300	-	-	22.24
3	150	HNO_3_	10	76.77
4	150	HNO_3_	30	90.36

**Table 5:** Central composite experimental design and response values.

Run	Sulfuric Acid Concentration (g/L)	Solid Content (%)	Microwave Power (W)	HNO_3_ Concertation (g/L)	Process Time (min)	R_cu_ (%)	R_zn_ (%)
	A	B	C	D	E	-	-
1	100	10	400	15	15	32.70	98.82
2	200	10	400	15	6	25.55	80.37
3	100	20	400	15	6	20.88	84.16
4	200	20	400	15	15	25.92	80.96
5	100	10	800	15	6	25.23	85.28
6	200	10	800	15	15	34.29	81.11
7	100	20	800	15	15	21.84	87.08
8	200	20	800	15	6	23.85	88.33
9	100	10	400	25	6	24.41	86.36
10	200	10	400	25	15	33.94	88.81
11	100	20	400	25	15	21.42	76.69
12	200	20	400	25	6	22.99	74.99
13	100	10	800	25	15	29.66	69.73
14	200	10	800	25	6	28.63	70.66
15	100	20	800	25	6	21.60	70.37
16	200	20	800	25	15	28.87	70.76
17	50	15	600	20	10.5	21.40	69.05
18	250	15	600	20	10.5	39.35	74.91
19	150	5	600	20	10.5	34.82	78.51
20	150	25	600	20	10.5	21.73	74.94
21	150	15	200	20	10.5	22.18	86.16
22	150	15	1000	20	10.5	23.61	89.74
23	150	15	600	10	10.5	22.68	85.50
24	150	15	600	30	10.5	25.38	89.58
25	150	15	600	20	1.5	19.97	86.39
26	150	15	600	20	19.5	27.27	86.51
27	150	15	600	20	10.5	24.62	89.57
28	150	15	600	20	10.5	24.68	82.82
29	150	15	600	20	10.5	24.84	88.07
30	150	15	600	20	10.5	24.43	81.85
31	150	15	600	20	10.5	22.80	87.04
32	150	15	600	20	10.5	23.56	84.88

**Table 6:** Analysis of variance for copper recovery.

Source	Sum of Squares	Degree of Freedom	Mean Square	*F*-Value	*p*-Value	Explanation
Model	1.111 × 10^−3^	9	1.235 × 10^−4^	26.15	<0.0001	Significant
A	2.858 × 10^−4^	1	2.858 × 10^−4^	60.55	<0.0001	
B	4.362 × 10^−4^	1	4.362 × 10^−4^	92.39	<0.0001	
C	1.092 × 10^−5^	1	1.092 × 10^−5^	2.31	0.1425	
D	5.879 × 10^−6^	1	5.879 × 10^−6^	1.25	0.2765	
E	2.187 × 10^−4^	1	2.187 × 10^−4^	46.32	<0.0001	
BC	1.562 × 10^−5^	1	1.562 × 10^−5^	3.31	0.0825	
CD	1.986 × 10^−5^	1	1.986 × 10^−5^	4.21	0.0523	
A^2^	7.701 × 10^−5^	1	7.701 × 10^−5^	16.31	0.0005	
B^2^	4.885 × 10^−5^	1	4.885 × 10^−5^	10.35	0.0040	
Residual	4.721 × 10^−4^	22	4.721 × 10^−6^			
Lack of Fit	9.387 × 10^-−5^	17	9.387 × 10^−5^	2.76	0.1324	Not-significant
Pure Error	9.987 × 10^−6^	5	9.987 × 10^−6^			
Total	1.215 × 10^−3^	31				

**Table 7:** Analysis of variance for zinc recovery.

Source	Sum of Squares	Degree of Freedom	Mean Square	*F*-Value	*p*-Value	Explanation
Model	1105.92	9	122.88	5	0.001	Significant
A	4.84	1	4.84	0.2	0.6615	
B	50.82	1	50.82	2.07	0.1644	
C	68.90	1	68.90	2.81	1081	
D	201.55	1	201.55	8.21	0.0090	
E	7.79	1	7.79	0.32	0.5790	
BC	139.94	1	139.94	5.70	0.0260	
CD	114.61	1	114.61	4.67	0.0419	
A^2^	379.85	1	379.85	15.47	0.0007	
B^2^	169.10	1	169.10	6.88	0.0155	
Residual	540.35	22	24.56			
Lack of Fit	494.19	17	29.07	3.15	0.1045	Not-significant
Pure Error	46.17	5	9.23			
Total	1646.27	31				
